# Trunk Muscle Activity during Drop Jump Performance in Adolescent Athletes with Back Pain

**DOI:** 10.3389/fphys.2017.00274

**Published:** 2017-05-04

**Authors:** Steffen Mueller, Josefine Stoll, Juliane Mueller, Michael Cassel, Frank Mayer

**Affiliations:** University Outpatient Clinic, Sports Medicine and Sports Orthopaedics, University of PotsdamPotsdam, Germany

**Keywords:** SEMG-pattern, back pain, pre-activity, drop jump, neuromuscular, trunk, performance, young athletes

## Abstract

In the context of back pain, great emphasis has been placed on the importance of trunk stability, especially in situations requiring compensation of repetitive, intense loading induced during high-performance activities, e.g., jumping or landing. This study aims to evaluate trunk muscle activity during drop jump in adolescent athletes with back pain (BP) compared to athletes without back pain (NBP). Eleven adolescent athletes suffering back pain (BP: m/f: *n* = 4/7; 15.9 ± 1.3 y; 176 ± 11 cm; 68 ± 11 kg; 12.4 ± 10.5 h/we training) and 11 matched athletes without back pain (NBP: m/f: *n* = 4/7; 15.5 ± 1.3 y; 174 ± 7 cm; 67 ± 8 kg; 14.9 ± 9.5 h/we training) were evaluated. Subjects conducted 3 drop jumps onto a force plate (ground reaction force). Bilateral 12-lead SEMG (surface Electromyography) was applied to assess trunk muscle activity. Ground contact time [ms], maximum vertical jump force [N], jump time [ms] and the jump performance index [m/s] were calculated for drop jumps. SEMG amplitudes (RMS: root mean square [%]) for all 12 single muscles were normalized to MIVC (maximum isometric voluntary contraction) and analyzed in 4 time windows (100 ms pre- and 200 ms post-initial ground contact, 100 ms pre- and 200 ms post-landing) as outcome variables. In addition, muscles were grouped and analyzed in ventral and dorsal muscles, as well as straight and transverse trunk muscles. Drop jump ground reaction force variables did not differ between NBP and BP (*p* > 0.05). Mm obliquus externus and internus abdominis presented higher SEMG amplitudes (1.3–1.9-fold) for BP (*p* < 0.05). Mm rectus abdominis, erector spinae thoracic/lumbar and latissimus dorsi did not differ (*p* > 0.05). The muscle group analysis over the whole jumping cycle showed statistically significantly higher SEMG amplitudes for BP in the ventral (*p* = 0.031) and transverse muscles (*p* = 0.020) compared to NBP. Higher activity of transverse, but not straight, trunk muscles might indicate a specific compensation strategy to support trunk stability in athletes with back pain during drop jumps. Therefore, exercises favoring the transverse trunk muscles could be recommended for back pain treatment.

## Introduction

Back pain (point) prevalence in adolescent athletes is reported at a rate of 8–20%, with a relevant increase beginning around age 14 and featuring sport-specific differences (Schmidt et al., 2014; Müller et al., 2017). Consequently, back pain can be considered a relevant risk factor in the careers of young elite athletes.

In the context of back pain, great emphasis has been placed on the importance of trunk stability, especially in situations requiring compensation of repetitive, intense loading induced during high-performance activities, e.g., jumping or landing (Cholewicki et al., 2000; Borghuis et al., 2008; Simons and Bradshaw, 2016). In etiology, repetitive micro-trauma and insufficiency of the muscle-tendon complex based on an inadequate neuromuscular and postural control, and a reduced maximum strength capacity in addition to trunk muscle fatigue during dynamic loading are discussed as an explanatory model (George and Delitto, 2002; Sassmannshausen and Smith, 2002; Standaert, 2002, 2008; Trainor and Wiesel, 2002; Bono, 2004; Trainor and Trainor, 2004; Lawrence et al., 2006). In particular, trunk muscle forces providing stability are considered meaningful in counteracting high-impact loading during high-intensity activities (Kibler et al., 2006; Borghuis et al., 2008; Larivière et al., 2015; Prieske et al., 2016). When compensating high loading, a reduced trunk strength capacity as well as delayed muscle onset, increased co-contractions, and increased SEMG variability has been shown in back pain patients (Cholewicki et al., 2000; Radebold et al., 2000, 2001; Marras et al., 2005). In a recent systematic review, Abboud et al. could moreover show evidence for back pain related decreased erector spinae and increased external obliquus muscle reflex amplitudes (Abboud et al., 2016). In addition, trunk strength capacity is considered essential for the compensation of external forces and loads in young and adult athletes (Kibler et al., 2006; Zazulak et al., 2006, 2007; Wirth et al., 2016).

As reported in previous studies, different types of sports reveal specific demands on core stability (Helge and Kanstrup, 2002; McGregor et al., 2004; Iwai et al., 2008; Baur et al., 2010; Mueller et al., 2012). Nevertheless, Kibler et al. (2006) described the role of core stability for all types of sports, whether running, throwing or jumping tasks. High-impact forces acting on the trunk are reported in judo, rowing, weight lifting, (rhythmic) gymnastics, and jumping (Liemohn et al., 2005; Peate et al., 2007; Hibbs et al., 2008; Ripamonti et al., 2008). Repetitive loading with large components of translation, rotation and reclination movements are believed to result in stress of the structures involved (Hutchinson, 1999; Sassmannshausen and Smith, 2002; Adirim and Cheng, 2003; Jones et al., 2005). It could be shown that athletic tasks like running, hopping, jumping and landing increase the impact forces that need to be compensated (Dufek and Bates, 1990; Keller et al., 1996; Simons and Bradshaw, 2016). Simons and Bradshaw (2016) reported an additional loading of the trunk with up to eight times the body weight during repetitive hopping or drop landing. The importance of trunk strength capacity was recently reported as beneficial not only for compensating loading and stabilizing the trunk, but also for enhancing athletic performance (Kibler et al., 2006; Zazulak et al., 2006, 2007). Furthermore, Zazulak et al. (2007) reported an association between trunk muscle activity and lower limb kinematics during landing tasks. Decreased neuromuscular activity is attributed to higher knee valgus, increasing injury risk at the knee. Hence, an inadequate (neuromuscular) compensation strategy is discussed as a common cause of overloading and injury. As a possible consequence, there is a need to identify relevant trunk muscles that must necessarily be addressed in injury and overload prevention. Nevertheless, the role of back pain as one factor influencing neuromuscular activity of the trunk-encompassing muscles has not been clarified.

Therefore, this study aims to evaluate trunk muscle activity during drop jump (DJ) in adolescent athletes with back pain compared to their healthy counterparts. An altered neuromuscular activity strategy driven by higher ventral and reduced dorsal muscle SEMG amplitudes in athletes suffering from back pain while performing and compensating high-impact loading during drop jumps is expected compared to healthy athletes.

## Materials and methods

### Subjects

Twenty-two adolescent athletes (*n* = 11 with back pain, BP; *n* = 11 gender and age matched athletes without back pain, NBP) were enrolled in the study from different sports (BP: *n* = 8 canoeing/rowing, *n* = 2 triathlon, *n* = 1 wrestling; NBP: *n* = 9 canoeing/rowing, *n* = 2 triathlon). Age below 18 years and affiliation with the organized training system for elite athletes served for inclusion criteria, and acute infection, contraindications for exercise or pain other than BP served as exclusion criteria. BP was defined as current back pain intensity assessed with a visual analog scale (VAS: 0–10 cm; 0 = no pain, 10 = maximum imaginable back pain). All athletes reporting VAS ≥ 2.0 cm were assigned to BP (Nelson-Wong et al., 2012). This type of questionnaire is described as valid for the use of subjective pain assessment in adolescents (Kropp, 2004; Merati et al., 2004). Anthropometrics for BP and NBP are detailed in Table 1. This study was carried out in accordance with the recommendations of the European Community Good Clinical Practice (EC-GCP), approved by the University Potsdam Ethical Committee. All participants and their legal guardians were informed of the study and the specific testing procedures in a personal conversation with the principle investigator and through written study information during their stay at the University Outpatient Clinic. Before voluntary participation in the study, the legal guardian and the adolescent participant provided written informed consent in accordance with the Declaration of Helsinki.

**Table 1:** **Characteristics of adolescent athletes (anthropometric and training data) with (BP) and without (NBP) back pain**.

**Group**	***n* (f/m)**	**Age [years]**	**Height [cm]**	**Weight [kg]**	**tr. volume [h/week]**	**Back pain VAS [cm]**
BP	7/4	15.9 ± 1.3	176 ± 11	68 ± 11	12.4 ± 10.7	3.2 ± 1.4
NBP	7/4	15.5 ± 1.3	174 ± 7	67 ± 8	14.9 ± 9.5	0 ± 0

### Procedures

A cross-sectional study design was used to evaluate drop jump performance in young athletes with and without back pain. The test protocol started with a medical check-up to ensure that all participants were suitable for the upcoming jumping tests. In addition, anthropometric data, training history and subjective back pain intensity (visual analog scale VAS) were assessed. Afterwards, all participants were prepared for SEMG analysis of the trunk muscles. Following this, all athletes underwent a general physical warm-up of at least 5 min prior testing. For SEMG normalization, the maximum isometric voluntary contraction (MIVC) of trunk flexion and extension was measured using an isokinetic dynamometer (Contrex MJ/TP, Physiomed AG, Schnaittach, Germany). After 1 min. of trunk extension/flexion warm-up and a practice trial for maximum isometric trunk flexion and for trunk extension on the dynamometer, the test was executed for 5 s each time. Participants were fixed to the dynamometer in a standing position at the lower leg and the knee, and then additionally with 2 non-stretching belts at the hip and upper body. Measurement position was defined in a middle position at 17.5° trunk flexion. Further details for the positioning could be seen elsewhere (Mueller et al., 2014). Then, complex motor performance was assessed with drop jumps (DJ). Initial instruction was followed by a demonstration and one practice trial before jump measurements were performed. Three repetitions were always captured for DJ.

### Ground reaction force

Drop jumps were performed from a 20 cm-high box onto a ground reaction force (GRF) plate (Amti OR6-6, Advanced Mechanical Technology, Inc., Watertown USA). Participants were instructed to drop onto the plate and jump as fast and high as possible from the plate, finally landing stably on the plate. No restrictions on arm movement were given. Ground contact time (Ct: [ms]), jump time (Jt: [ms]), peak force at take-off (Fz: [N]) and the performance index (Pi: [m/s]; formula: performance index = jump height/contact time) were calculated as the mean of 3 drop jumps GRF and act as secondary variables (Prieske et al., 2013).

### Trunk muscle activity

**Figure 1:**
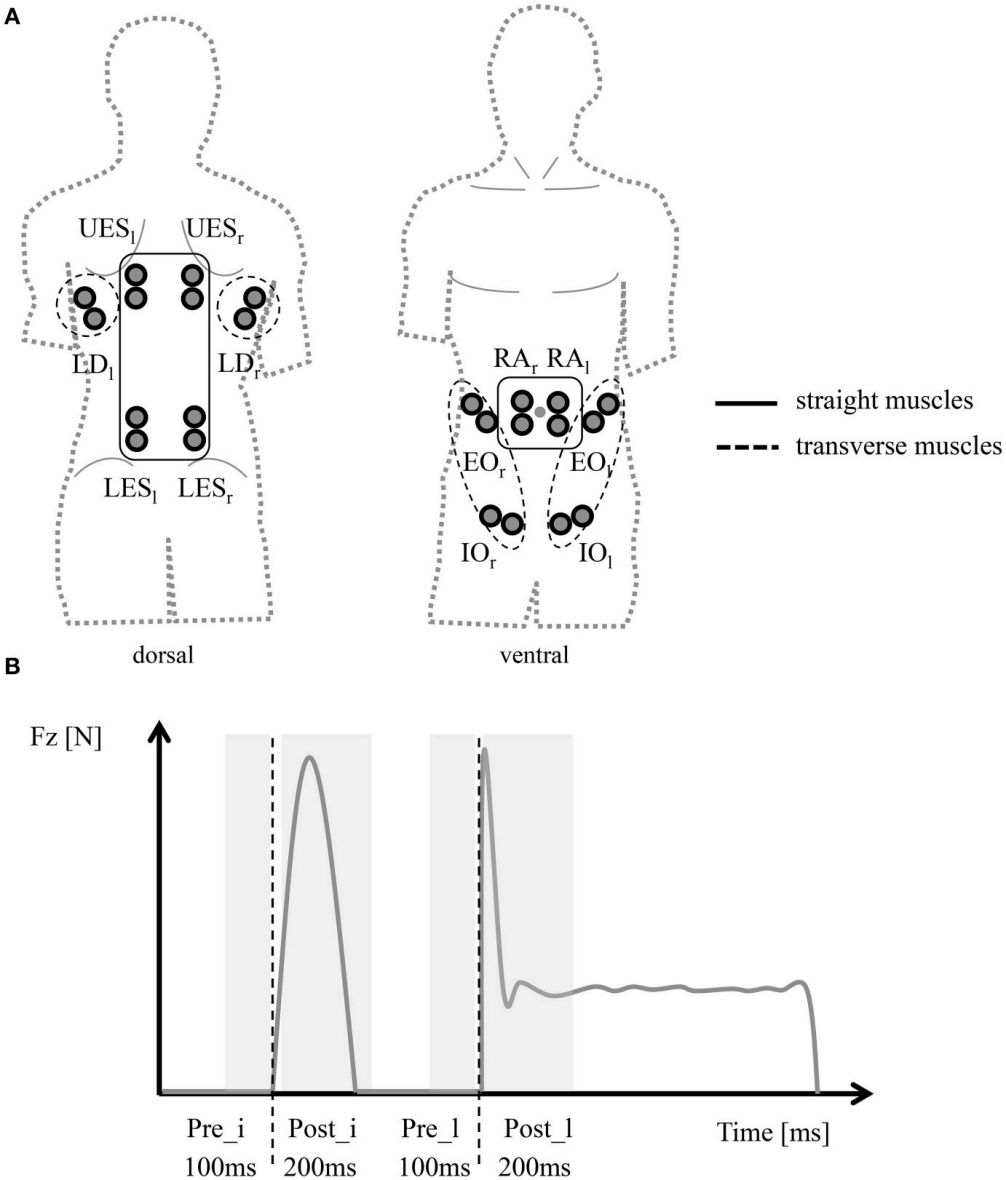
**Methodological SEMG setup. (A)** Bilateral 12-lead SEMG setup framing groups of transverse and straight trunk muscles. **(B)** Time windows for SEMG-RMS analysis [100 ms pre- (Pre_i), 200 ms post- (Post_i) initial ground contact; 100 ms pre- (Pre_l), 200 ms post- (Post_l) drop jump landing] triggered by ground reaction force (Fz) signal.

Muscular activity of the trunk was assessed using a bilateral 12-lead SEMG (Radebold et al., 2000) (Figure 1A): Mm. rec. abd. (RA), obl. ext. abd. (EO), obl. int. abd (IO); Mm. erec. spinae thoracic (T9; UES)/lumbar (L3; LES), latis. dorsi (LD). The location of the electrodes was carefully determined according to Radebold et al. (2000). Before electrodes (AMBU Medicotest, Denmark, Type N-00-S, interelectrode distance: 2 cm) were applied, the skin was shaved and slightly roughened to remove surface epithelial layers and control skin resistance (<5 kΩ). The longitudinal axes of the electrodes were placed in line with the underlying muscle fibers and checked for minimum cross-talk by inspection during the initial muscle tests.

For SEMG data analysis, muscular activity was analyzed using a bilateral and bipolar surface telemetric SEMG (band-pass filter: 5–500 Hz, gain: 5.0, overall gain: 2,500, sampling frequency: 4,000 Hz, RFTD32, myon AG, Baar, CH). No additional filter was applied post processing. The signal was rectified and averaged before calculation of the outcome measures. SEMG amplitudes (rout mean square RMS: [%]) were normalized to the isometric maximum voluntary contractions (MIVC). Mean amplitudes for the left and right side were calculated separately and both sides were averaged for each muscle and analyzed in 4 time windows [100 ms pre- (Pre_i), 200 ms post- (Post_i) initial ground contact; 100 ms pre- (Pre_l), 200 ms post- (Post_l) drop jump landing], triggered manually by an experienced investigator using the ground reaction force signal, as SEMG measurements (Figure 1B). Jumping trials showing artifacts (movement) were not considered for further analysis (Software: IMAGO process master, LabView®-based, pfitec, biomedical systems, Endingen, Germany).

As SEMG outcome variables, the SEMG-RMS measurements [%] for all 12 muscles were computed (secondary outcomes). After calculation of the mean across all 4 time windows, muscles were grouped and analyzed in ventral (grouping of RA, IO, EO) and dorsal (grouping of LD, UES, LES) muscles, as well as straight (grouping of RA, UES, LES) and transverse (grouping of IO, EO, LD) trunk muscles. The SEMG-RMS for the grouped ventral muscles was defined as primary outcome all other variables as secondary outcomes.

### Statistical analysis

All non-digital data were documented in a handwritten case report form and transferred to a database for further statistical analysis (JMP Statistical Software Package 9, SAS Institute®). For all data, a plausibility check was performed. Implausible values (range check) were compared with the raw data and corrected/recalculated (<1%), if necessary. After data was tested for normality (Shapiro-Wilk-Test) descriptive statistics (mean ± SD) was followed by un-paired *t*-test to account for differences between groups (BP/NBP) (α = 0.05).

## Results

### Ground reaction force

The GRF measurements did not differ between NBP and BP (*p* > 0.05). Overall, athletes showed a ground contact time of 290 ± 71 ms, a mean jump time of 434 ± 49 ms, a peak force during take-off phase of 2,756 ± 539 N and a performance index of 0.83 ± 0.21 m/s. Group results (NBP/BP) for jump performance are detailed in Table 2.

**Table 2:** **Drop jump performance [ground contact time (Ct; ms); jump time (Jt; ms); peak force at take-off (Fz; N); performance index (Pi; m/s)] in athletes with (BP) and without (NBP) back pain (mean ± SD)**.

**Group**	**Contact time (Ct, [ms])**	**Jump time (Jt, [ms])**	**Peak force (Fz, [N])**	**Performance index (Pi, [m/s])**
BP	279 ± 60	427 ± 51	2677 ± 333	0.827 ± 0.188
NBP	302 ± 82	441 ± 49	2835 ± 697	0.837 ± 0.232

### Trunk muscle activity

**Figure 2:**
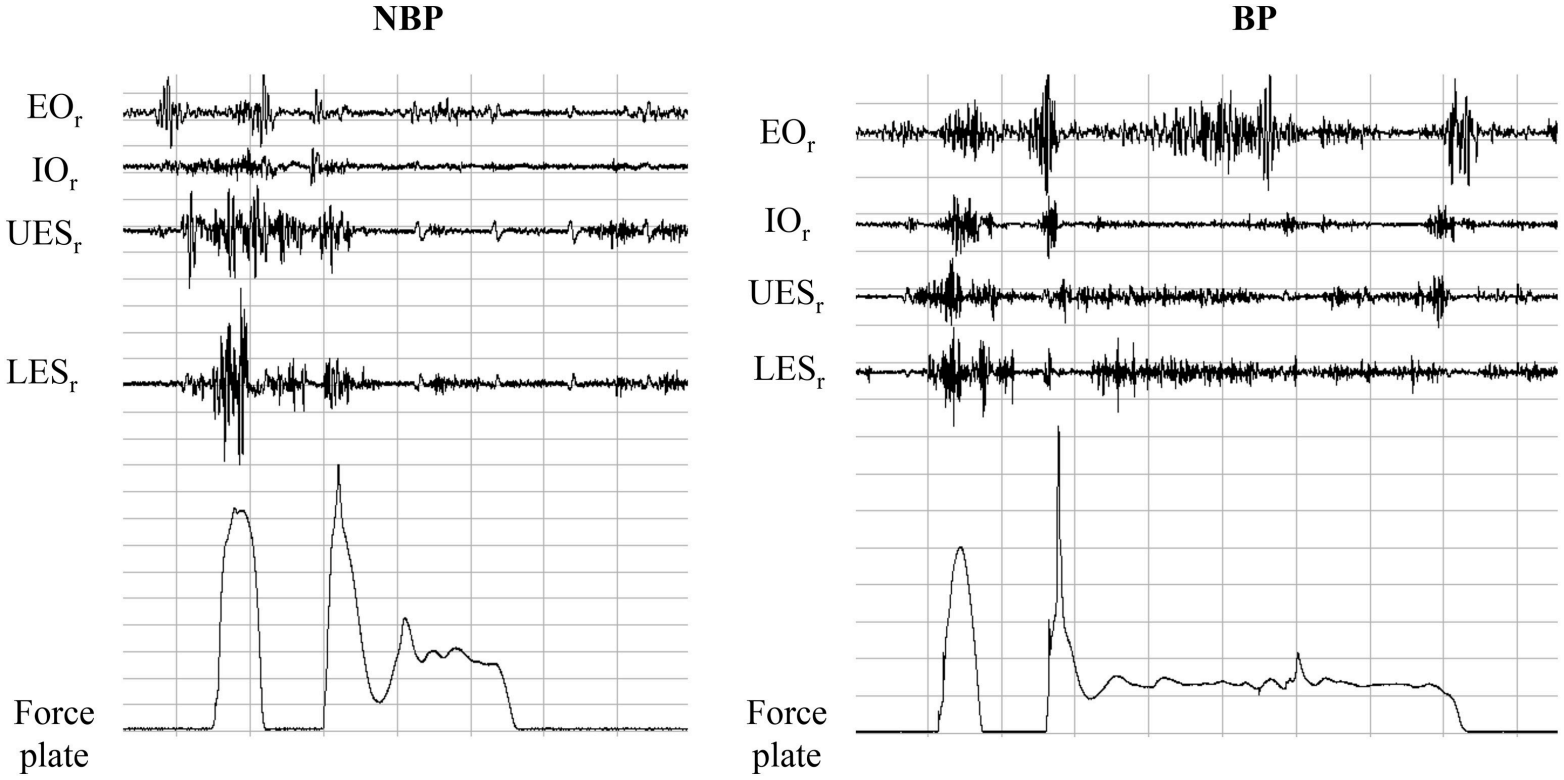
**In an exemplary way SEMG raw signals, for Mm. obl. ext. abd. (EO), obl. int. abd (IO); Mm. erec. spinae thoracic (T9; UES)/lumbar (L3; LES), and for ground reaction force signal (Fz) for one subject with (BP) and without (NBP) back pain, are shown**.

**Figure 3:**
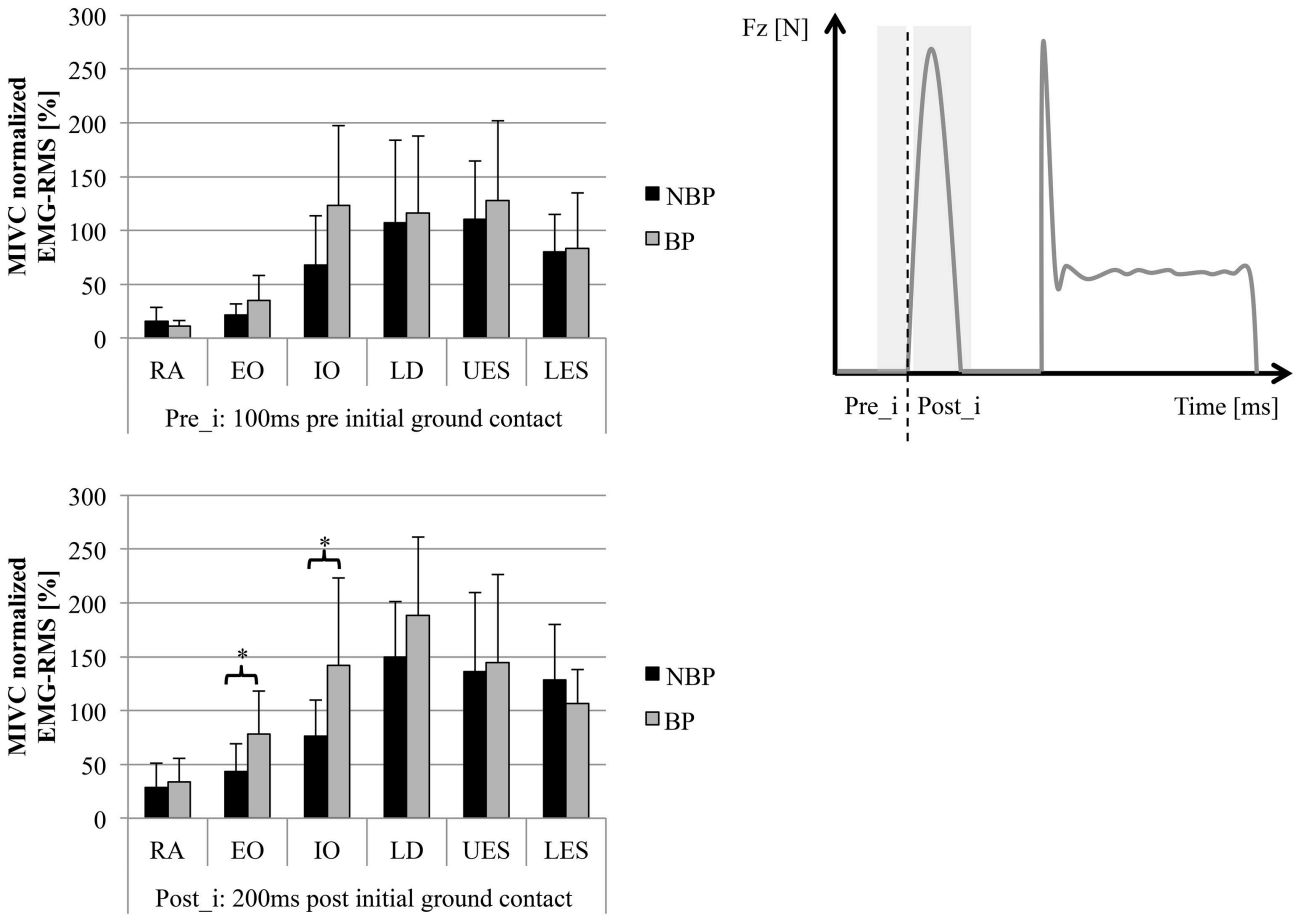
SEMG-RMS (normalized MIVC [%]) for 6 trunk muscles (average of left/right sides) for pre- (Pre_i) and post- (Post_i) initial ground contact phase (mean ± SD; ^*^p < 0.05). Muscles: Mm. rec. abd. (RA), obl. ext. abd. (EO), obl. int. abd (IO); Mm. erec. spinae thoracic (T9; UES)/lumbar (L3; LES), latis. dorsi (LD).

**Figure 4:**
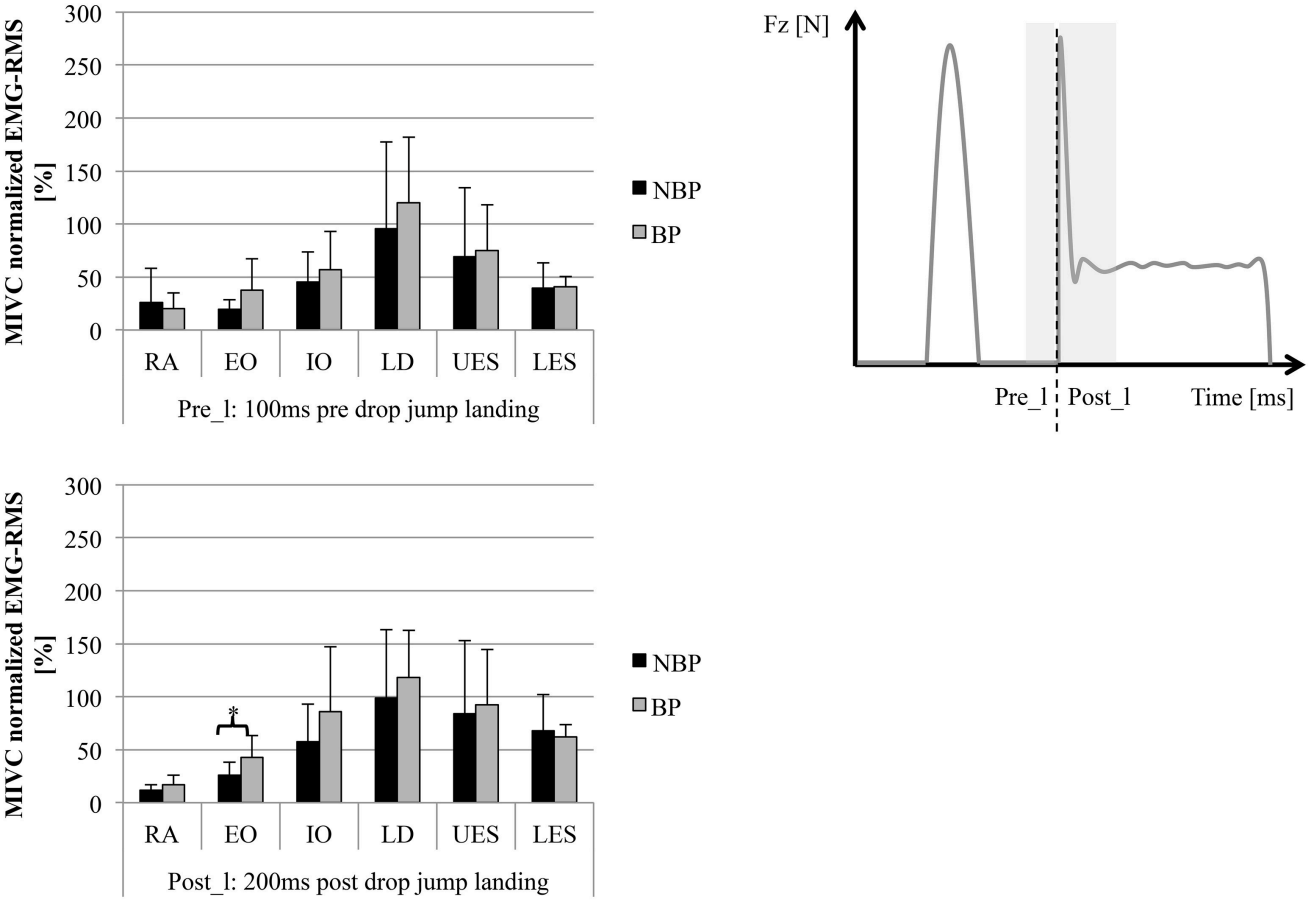
SEMG-RMS (normalized MIVC [%]) for 6 trunk muscles (average of left/right sides) for pre- (Pre_l) and post- (Post_l) initial drop jump landing (mean ± SD; ^*^p < 0.05). Muscles: Mm. rec. abd. (RA), obl. ext. abd. (EO), obl. int. abd (IO); Mm. erec. spinae thoracic (T9; UES)/lumbar (L3; LES), latis. dorsi (LD).

In the pre-activity phase, 100 ms pre-initial ground contact, SEMG-RMS ranged from 15 ± 13 to 110 ± 54% in NBP and 11 ± 5 to 128 ± 74% in BP without significant group differences. SEMG-RMS for the 200 ms post-initial ground contact ranged from 28 ± 23 to 149 ± 52% in NBP and 34 ± 22 to 188 ± 73% in BP. BP revealed higher SEMG activity compared to NBP, except for LES, and was statistically significant for EO and IO (*p* = 0.033/0.027; *t* = 2.29/2.39) (Figures 2, 3). In the second pre-activity phase, 100 ms before drop jump landing, LD showed the highest activity (NBP: 95 ± 182%; BP: 120 ± 62%) in both groups. Nevertheless, statistically significant differences between groups were not present. After landing, 200 ms post-drop jump landing, SEMG-RMS ranged from 12 ± 5% to 99 ± 64% in NBP and 17 ± 9% to 118 ± 44%. Group differences were only present for EO, with BP showing higher values compared to NBP (*p* = 0.041; *t* = 2.20) (Figure 4). Overall, EO and IO presented a 1.26- to 1.93-fold higher SEMG-RMS in BP compared to NBP for all time windows analyzed. SEMG-RMS for RA, UES, LES, and LD did not differ between groups in the four time windows analyzed. For groups, all muscles and analyzed time phases normalized SEMG-RMS values are shown in Table 3.

**Table 3:** **SEMG-RMS (normalized MIVC [%]) for 6 trunk muscles (average of left/right sides) for pre- (Pre_i) and post- (Post_i) initial ground contact phase and for pre- (Pre_l) and post- (Post_l) initial drop jump landing (mean ± SD)**.

**Group**	**Phase**	**RA**	**EO**	**IO**	**LD**	**UES**	**LES**
BP	Pre_i	11 ± 5	35 ± 23	124 ± 74	116 ± 71	128 ± 74	84 ± 51
NBP	Pre_i	15 ± 13	21 ± 11	68 ± 45	108 ± 76	110 ± 54	80 ± 35
BP	Post_i	34 ± 22	78 ± 40	142 ± 81	188 ± 73	144 ± 82	106 ± 31
NBP	Post_i	28 ± 23	44 ± 26	76 ± 34	149 ± 52	136 ± 73	128 ± 51
BP	Pre_l	20 ± 15	38 ± 30	57 ± 36	120 ± 62	75 ± 43	41 ± 10
NBP	Pre_l	26 ± 32	20 ± 9	45 ± 29	95 ± 82	69 ± 65	40 ± 24
BP	Post_l	17 ± 9	43 ± 21	86 ± 61	118 ± 44	93 ± 52	62 ± 12
NBP	Post_l	12 ± 5	26 ± 12	57 ± 36	99 ± 64	84 ± 69	68 ± 34

*Muscles: Mm. rec. abd. (RA), obl. ext. abd. (EO), obl. int. abd (IO); Mm. erec. spinae thoracic (T9; UES)/lumbar (L3; LES), latis. dorsi (LD)*.

**Figure 5:**
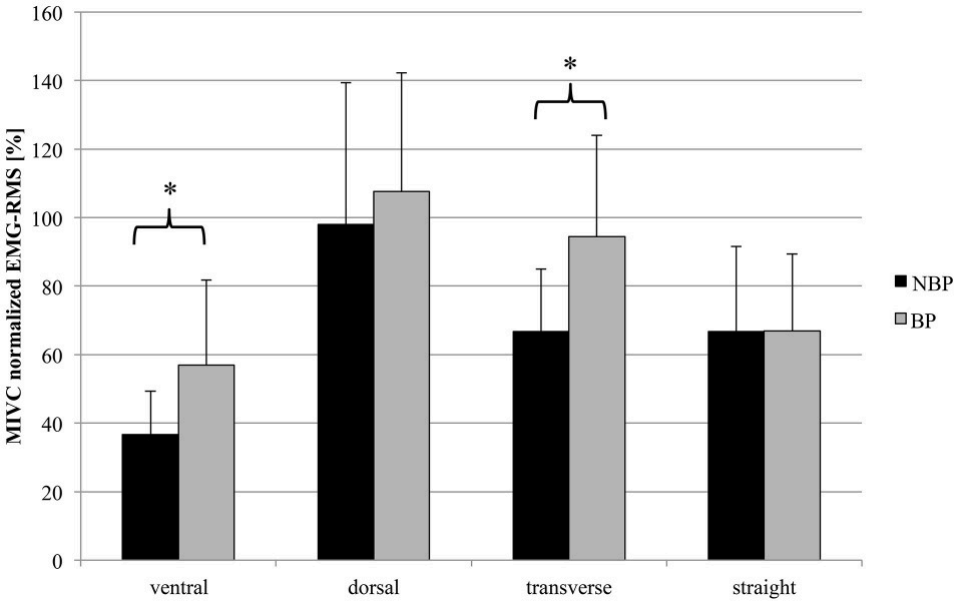
Mean SEMG-RMS (normalized MIVC [%]) for muscle groups (ventral vs. dorsal; transverse vs. straight) across total drop jump performance (mean ± SD; ^*^p < 0.05). Muscles: Mm. rec. abd. (RA), obl. ext. abd. (EO), obl. int. abd (IO); Mm. erec. spinae thoracic (T9; UES)/lumbar (L3; LES), latis. dorsi (LD).

The muscle group analysis over all 4 time window showed statistically significantly higher SEMG-RMS for BP in the ventral (*p* = 0.031; *t* = 2.34; power = 0.6021) and transverse muscles (*p* = 0.020; *t* = 2.55), with BP showing higher amplitudes compared to NBP (Figure 5).

## Discussion

Altered neuromuscular activity in back pain patients compared to healthy subjects is known (Radebold et al., 2001; Maaswinkel et al., 2016). This study aimed to evaluate neuromuscular activity of the trunk muscles during high-impact loading represented by drop jump performance in adolescent athletes with back pain compared to healthy counterparts. The main findings of the investigation are an altered neuromuscular activation pattern in adolescent athletes with back pain, with increased SEMG amplitudes for the M. obl. ext. abd (EO) and M. obl. int. abd (IO) especially during reactive ground contact (Post_i). Regarding the total jumping cycle the abdominal and transverse muscle groups showed increased SEMG amplitudes with similar absolute drop jump performance measurements.

There is existing evidence that athletic performance and function is an outcome of the appropriate (neuromuscular and kinetic) coordination of body segments (Kibler et al., 2006). Back pain is often discussed as an influencing factor, but, despite the existence of back pain, the adolescent BP athletes do not show a reduction in (drop) jump performance capacity. Jump performance alone might therefore not serve as a valid indicator for deficits in adolescent athletes with back pain. In this context, the level of back pain and chronification might play a relevant role. It could also be speculated that high pain levels compared to our cohort might show reduced performance as described in other papers (Balagué et al., 2012; Bauer et al., 2015).

However, in terms of trunk muscle activity, BP is associated with an altered neuromuscular activity level as reported previously (Radebold et al., 2001; Nelson-Wong and Callaghan, 2010; Abboud et al., 2016; Maaswinkel et al., 2016). A decreased muscle activity for the dorsal muscle group (e.g., erector spinae), as reported by Ramprasad et al., and Shenoy et al., for patients with back pain, could not be supported by our data (Ramprasad et al., 2010; Shenoy et al., 2013). But, increased muscle activity in the abdominal and transverse muscle groups correspond to higher co-contractions, already presented in other trunk loading experiments (Radebold et al., 2001; Nelson-Wong and Callaghan, 2010). Furthermore, Liebetrau et al. could present in their musculoskeletal model that delayed abdominal muscle reflex lead to a reduced trunk stability. Information's about detailed association for pain and function (higher pain—higher functional deficits) are rare since experimental studies mainly remain to the dichotomous comparisons of pain to identify functional differences (Liebetrau et al., 2013; Schinkel-Ivy et al., 2013; Abboud et al., 2016). To compensate delayed reflex activity, higher activation amplitudes of the abdominal muscles are valid (Liebetrau et al., 2013). Transferring this to drop jumps, it could be speculated that the shown increased muscle activity (Mm obl. abd. intern./extern.) serves as a compensation strategy to enhance core stability and protect the trunk from further negative loading (Kibler et al., 2006; Hibbs et al., 2008; Prieske et al., 2015; Wirth et al., 2016). It is also known that the stability provided by the trunk muscles is meaningful in counteracting single and repetitive loading during high-intensity performance (Kibler et al., 2006).

The combination of a similar (jump) performance and yet increased neuromuscular trunk activity level in adolescent BP athletes compared to NBP athletes still appears controversial. It could be speculated that BP athletes present a less efficient jump performance execution. Otherwise, the additional muscle activation might show the functional adaptive response in athletes supporting an appropriate protection from overloading (Maaswinkel et al., 2016). Therefore, drop jumps could serve as a suitable test situation to analyze alterations in the neuromuscular trunk activity of athletes with and without back pain. Additionally, SEMG activity might reflect the compensation capacity for repetitive high loading. It remains to be seen whether this might not only serve to identify athletes suffering from back pain, but also athletes at risk for developing it. In addition, this analysis might allow a valid evaluation of athletes in order for them to return to sports after a period of back pain. Nevertheless, these points of discussion need further verification.

Consequently, optimizing neuromuscular core stability is considered beneficial for protection against repetitive and excessive overloading of the trunk (Kibler et al., 2006; Borghuis et al., 2008; Saragiotto et al., 2016; Wirth et al., 2016). In line with the results presented here, core stability exercises (with additional repetitive loading) addressing the complex interaction of transverse, straight, ventral and dorsal muscles in adolescent athletes with back pain should be preferred (Pedersen et al., 2004; Saragiotto et al., 2016).

Certain limitations have to be considered when interpreting the results. Acute but no average pain was assessed and association of pain with outcome variables is not presented since correlation analysis would not be valid for the existing skewed pain distribution (no subjects with pain intensity between 0 and 2 VAS). SEMG normalization is often used to acquire comparable values between different individuals and groups, but contains some crucial points. The pain might influence the MIVC measurement used, since the intended 100% activation might not be reached. This could lead to reduced strength values and a systematic overestimation of the normalized SEMG amplitudes during the jumps for the patients (Marras et al., 2005). Our data showed for the MIVC measurement in extension and flexion (18/23%) lower strength values for BP. But, the higher (BP) activity during jumping was only for the flexor muscles, but not for the others present. Therefore, considering this limitation, the SEMG method and normalization procedure used seem valid. Furthermore, the highly standardized dynamometer-based MIVC extension and flexion test might not challenge all ventral/dorsal or straight/transverse muscles identically (Iida et al., 2011). This should be noted to put the normalized SEMG values (e.g., LD > 100%) into perspective. The SEMG setup used has been shown as valid in previous studies, but the possibility of a bit of cross-talk between muscles cannot be totally denied (Radebold et al., 2001). Concerning the investigated athletes performing mainly rowing, the applicability to other sports needs to be proven in future investigations. Finally, only the current, but not the average back pain intensity across days or weeks was assessed and might have influenced the results.

In conclusion, adolescent athletes with moderate back pain intensity are capable of presenting (jump) performance comparable to that of their healthy counterparts. Higher activity of the transverse, but not the straight, trunk muscles indicates a specific compensation strategy to support trunk stability in athletes with back pain during drop jumps. For prevention and therapy, specific sensorimotor exercises addressing the transverse trunk muscles with e.g., 3-dimensional loading situations might be beneficial.

## Author contributions

The author contributions are distributed as followed: conception or design of the work (SM, JM, FM), data acquisition (SM, JS, JM, MC), data analysis (SM, JM) and interpretation (SM, JM, FM); drafting (SM) or revising (JS, JM, MC, FM) the work; final approval of the version to be published (SM, JS, JM, MC, FM); agreement to be accountable for all aspects of the work (SM, JS, JM, MC, FM).

## Funding

This study was supported by a research grant from the National Institute of Sport Science of Germany (Bundesinstitut für Sportwissenschaft BISp: IIA 1-080126/09-13). We acknowledge the support of the Deutsche Forschungsgemeinschaft and Open Access Publishing Fund of University of Potsdam.

### Conflict of interest statement

The authors declare that the research was conducted in the absence of any commercial or financial relationships that could be construed as a potential conflict of interest. The handling Editor declared a past co-authorship with one of the authors FM and states that the process nevertheless met the standards of a fair and objective review.
